# Factors associated with anxiety in family caregivers of children with chronic diseases

**DOI:** 10.1186/s13030-018-0139-7

**Published:** 2018-12-12

**Authors:** Filiberto Toledano-Toledano, José Moral de la Rubia

**Affiliations:** 1 Unidad de Investigación en Medicina Basada en Evidencias, Hospital Infantil de México Federico Gómez Instituto Nacional de Salud, Dr. Márquez 162, Cuauhtémoc, 06720 México City, Mexico; 2 Facultad de Psicología, Universidad Autónoma de Nuevo León, Dr. Carlos Canseco, 110, Esq. Dr. Aguirre Pequeño, Col. Mitras Centro, 64460 Monterrey, México

**Keywords:** Family caregivers, Psychosocial factors, Family caregiver anxiety, Depression, Families, Pediatric chronic diseases, Family caregiver burden, Sociodemographic variables, Caregiver anxiety

## Abstract

**Background:**

Currently, information on factors associated with anxiety in family caregivers of children with chronic diseases is unavailable, indicating a significant gap in the literature. Therefore, this study aims to identify the psychosocial and sociodemographic variables associated with anxiety in family caregivers of children with chronic diseases.

**Methods:**

In 2018, a nonprobability sample of 446 family caregivers was recruited at the National Institute of Health in Mexico City. The participants completed a sociodemographic variable questionnaire, clinical questions, and 18 psychosocial assessment scales, including a scale to assess family caregiver anxiety.

**Results:**

Family caregiver anxiety was correlated with almost all psychosocial variables and one out of three clinical variables but with none of the sociodemographic variables. Furthermore, a multiple linear regression model with five psychosocial variables was established to predict family caregiver anxiety.

**Conclusions:**

Some psychosocial variables have effects on caregiver anxiety that are relevant for interventions. Clinical interventions should be implemented based on the psychosocial variables associated with family caregiver anxiety.

## Background

During the course of chronic disease in children, family caregivers actively participate in different areas of the child’s care, including assisting with the biomedical, physical, rehabilitation, psychological, family, social, and institutional health domains. In addition, caregivers are directly involved in long-term treatments, coordination of health services delivery, and management of the social, financial, and emotional challenges that accompany chronic diseases [1, 2]. Carrying out these responsibilities is necessary to provide chronically ill patients with the attention they need and to mitigate the lack of autonomy and independence experienced by the patient. However, these activities have physical, psychological, and economic repercussions for family caregivers [3, 4].

Among the main empirical findings regarding the effects of care on families of chronically ill children, research demonstrates an association between the attention and care provided to ill family members and caregiver anxiety [5, 6]. Anxiety is the most frequently occurring psychological disorder among family caregivers of children with complex diseases [7] and results from increased responsibilities and exhaustion [8]. Consequently, family caregivers exhibit behaviors that may pose a risk to their physical health, personal well-being, and mental health [9, 10].

Several studies have identified psychosocial and sociodemographic factors associated with anxiety in family caregivers of children with chronic diseases [1, 3, 5, 11]. The psychosocial factors identified in the literature include caregiver burden, quality of life, family functioning, health problems in daily life, parental stress, and depression [12–15]. In addition, the reports in the literature indicate that family caregivers with anxiety are likely to present with higher levels of depression and a greater caregiver burden [16–18] as well as a lower quality of life [4, 19], self-esteem [20], self-efficacy beliefs [21], activation and vitality levels [22], and resilience [23].

Regarding sociodemographic variables, recent studies have demonstrated that anxiety in family caregivers is associated with being a female caregiver [4, 24, 25], living in the same home as the patient [26], providing care for younger patients, receiving a poor prognosis [27], being a younger caregiver of ill children [28], being parents and caregivers of children with disabilities [29], psychosocial distress in family caregivers of children with cystic fibrosis [30], and having a low income [31]. In contrast, empirical evidence suggests that social support, appropriate stress management, and changes in one’s beliefs associated with the disease can help reduce anxiety in caregivers and facilitate adjustment to the disease [32–34]. Furthermore, the caregiver’s self-efficacy beliefs about completing care-related tasks, using positive coping styles, and being optimistic contribute to reducing anxiety and facilitating positive adjustment to the disease [21, 35–39]. Finally, high levels of education [40], subsidies received from the government and other institutions [41], and adequate family functioning [42] have positive effects on the caregiver’s adjustment and anxiety.

In summary, chronic pediatric diseases represent a challenge for families and particularly for family caregivers [23]. International research has provided a substantial amount of empirical evidence supporting the relationship between anxiety and numerous sociodemographic and psychosocial factors [13, 14, 27, 31]. However, few studies have integrated the psychosocial and sociodemographic perspectives to predict the multiple factors that increase the anxiety risk in family caregivers. Therefore, to contribute to the existing theoretical approaches and empirical evidence, the present research based on a Mexican sample was conducted with two objectives: (1) to describe the sociodemographic characteristics and anxiety levels of family caregivers of pediatric patients with chronic diseases in Mexico and (2) to evaluate the predictive power and relationship between anxiety in family caregivers in Mexico and 16 psychosocial variables (personal agency and empowerment, family support, social support, self-esteem, quality of life, primary caregiver burden, depression, negative coping, insecure attachment, parental stress, self-efficacy beliefs and optimism, familism or values reflecting obligation to family that supersedes attention to oneself, emotional well-being, internal locus of control, sociocultural tradition, and resilience), six sociodemographic variables (sex, age, education, and family income of the caregiver and sex and age of the child), and three clinical variables (child’s time of hospitalization, time since diagnosis, and clinical diagnosis).

In this study, we reviewed many studies aimed at predicting negative effects on caregivers from a psychosocial or epidemiological perspective, and we considered whether the variables included in this body of research were sufficient to achieve a high predictive power or large effect size. We even included two variables that have been rarely used in health psychology research but are very relevant in Mexican social psychology (familism and conservatism of sociocultural tradition), since they can act as protective factors against anxiety by providing structure for and greater support to the individual. Conversely, variables of temperament, genetics, and information processing, which are typical of biological or cognitive science approaches, were excluded from the approach. Therefore, the present research is a study with a psychosocial and sociodemographic perspectives.

## Methods

### Participants

In 2018, a cross-sectional study was conducted involving 446 family caregivers of children with chronic diseases hospitalized at the Federico Gómez Children’s Hospital of Mexico, National Institute of Health. Among the participants, 83% identified as female and 17% as male, and the average age was 32.23 years (*SD* = 8.65). The inclusion criteria for the present study required that the participants were over 18 years of age, were providing care for a child with a chronic disease who was hospitalized at the National Institute of Health, and read and signed an informed consent form prior to enrollment in the study. The study design was nonexperimental, cross-sectional, and ex post facto, with nonprobabilistic sampling [43].

### Instruments

Participants responded in writing to A Sociodemographic Variables Questionnaire (Q-SV) that was developed for research on family caregivers of children with chronic diseases [24]. This instrument is composed of 20 items that measure individual, familial, and caregiver factors, such as age, sex, and marital status. In addition, this instrument collects information on the child’s sex, age, diagnosis, and time of hospitalization, among other information. Additionally, they completed a battery of 18 self-reported instruments. Family caregiver anxiety was assessed using the Beck Anxiety Inventory (BAI). Detailed information regarding these instruments can be found in Table 1.

**Table 1:** Instruments Used to Evaluate the Psychosocial Variables Associated with Anxiety in Family Caregivers

Instrument	Author (year)	Number of items/response options	Factors	α
1. A Sociodemographic Variables Questionnaire (Q-SV)	Toledano-Toledano et al. [24]	20 items	Demographics, Medical, Family, and Sociocultural	
2.Beck Anxiety Inventory	Beck et al. [51]. Validated in a Mexican population by Robles et al. [53]	21/0 to 3	Subjective, Autonomic, Panic	.92
3.Scale of Personal Agency and Empowerment	Pick et al. [58]	42/1 (Never) to 4 (Always)	Personal Agency and Empowerment	.90
4.Quality of Life Inventory (Whoqol-Bref)	WHOQOL Group [59]. González-Celis and Sánchez-Sosa [60]	26/1 (Very dissatisfied) to 5 (Very satisfied)	Physical Health, Psychological Health, Social Relationships, and Environment	.90
5.Zarit Burden Interview	Zarit and Zarit [61]. Validated in a Mexican population by Alpuche et al. [62]	22/0 (Never) to 4 (Always)	Impact of Care on the Caregiver, Caregiver-Patient Interpersonal Relationship, and Self-Efficacy Expectations	.89
6.Beck Depression Inventory - II	Beck et al. [63] Validated in family caregivers of children with chronic diseases by Toledano-Toledano and Contreras-Valdez [49]	21/4	Affective, Cognitive, Somatic	.91
7.Coping Style Scale	Folkman and Lazarus [64] Validated in a Mexican population by Zavala et al. [65]	20/0 (Not at all) to 3 (Always or to a large degree)	Evasive Coping, Positive Reevaluation, Distancing, Denial, and Cognitive Analysis	.90
8.Scale of Attachment Styles	Márquez et al. [66]	21/1 (Totally disagree) to 5 (Totally agree)	Avoidant, Secure, and Anxious	.89
9.Parental Stress Scale	Oronoz et al. [67]	17/1 (Totally disagree) to 5 (Totally agree)	Stressors and Rewards	.89
10.Evaluation of Self-Efficacy Beliefs and Optimism	Rose et al. [68]	9/1 (Totally disagree) to 4 (Totally agree)	Self-Efficacy and Optimism/Pessimism	.88
11.Familism Scale	Lugo and Contreras [69]	18/1 (Totally disagree) to 5 (Totally agree)	Family Support, Family Interconnectedness, Family Honor, and Self-Subjugation to the Family	.88
12.Well-Being Index of the World Health Organization Scale	Bech et al. [70]	10/0 (Never) to 3 (All of the time)	Emotional Well-being	.89
13.Historic-Psycho-Socio-Cultural-Premises Scale (HSCPs).	Díaz-Guerrero [71]	33/1 (No, I do not agree) and 2 (Yes, I agree)	Traditional Family and Family in Transition	.88
14.Resilience Scale	Palomar-and Gómez [72] Validated in family caregivers of children with chronic diseases by Toledano-Toledano et al. [23]	43/1 (Totally disagree) to 4 (Totally agree)	Strength and Self-confidence, Social Competence, Family Support, Social Support and Structure	.95

### Procedure

This study was approved by the Research, Ethics, and Biosafety Commissions at the Hospital Infantil de México Federico Gómez National Institute of Health in Mexico City. We adhered to the ethics rules and considerations for research with humans currently in place in Mexico [44] as well as those outlined by the American Psychological Association [45]. Participation was voluntary, and all participants were informed of their rights under the Declaration of Helsinki prior to enrollment [46]. All family caregivers were informed of the objectives and scope of the investigation and of their research rights. Caregivers who agreed to participate in the study signed a consent letter. Consenting caregivers were given instructions and completed the questionnaires independently at their child’s hospital, and the battery of tests was administered individually.

The research team, which was composed of psychologists and social workers employed by the hospital, visited the waiting rooms of the different hospitalization services and spoke with potential participants. Then, the researchers met with each individual and provided information about the study, informed participants of their research rights, and gave them the informed consent form. Finally, the individuals who agreed to participate were given instructions on how to respond to the battery of self-reported instruments. The researchers took precautions to ensure that the participants understood that their participation was voluntary and that their responses would be kept confidential and anonymous. Each participant completed the sociodemographic questionnaire and battery of 18 instruments during one 30- to 50-min session. The data collection stage lasted approximately three months.

### Data analysis

The analyses for this study were conducted using SPSS (v.24, IBM Inc., USA) to describe the sample, frequencies, percentages (%), arithmetic means (*M*), and standard deviations (*SD*). Descriptive analyses (Ms, SDs, and ranges) were used to describe the distributions for each instrument included in this study. Pearson’s product-moment correlation coefficient (*r*) was used to investigate the associations between anxiety and the psychosocial, sociodemographic, and clinical variables (potential predictors). Point-biserial correlation was performed with dichotomous variables. The significance of the correlations was tested using Fisher’s Z transformation. A linear regression model to predict family caregiver anxiety was calculated with significant correlates using the backward-elimination method. The overall significance of the model was tested with the F-test, and the significance of each predictor was assessed using a t-test. The explanatory strength of the model was determined with the squared multiple correlation coefficient (*R*^2^). The multicollinearity of each predictor was evaluated based on the variance inflation factor (VIF). The residual analysis included the Durbin-Watson test to confirm the lack of autocorrelation between consecutive residuals (in order of sampling). Cook’s distance was calculated to identify influential values, and a histogram and Fisher’s skewness and kurtosis coefficients were used to assess whether the residuals were normally distributed. The distribution of residuals was considered normal if the histogram presented a bell-shaped curve and the symmetry and kurtosis coefficients were between − 0.5 and 0.5. The distribution was considered to be approaching normal if the histogram showed a bell-shaped curve, the coefficient of asymmetry was less than 1, and the kurtosis was less than 2 [47]. Following Cohen’s cut-off points, the effect size or strength of the association was interpreted as trivial when the standardized regression weights (β) or correlation coefficients (*r*) had values between − 0.099 and 0.099, low when between − 0.299 and − 0.10 or between 0.10 and 0.299, medium when between − 0.499 and − 0.30 or between 0.30 and 0.499, high when between − 0.699 and − 0.50 or between 0.50 and 0.699, very high when between − 0.899 and − 0.70 or between 0.70 and 0.899, and unitary when between − 1 and − 0.90 or between 0.90 and 1 [48].

## Results

### Sample description

The descriptive analysis results for the sociodemographic characteristics of the family caregivers, pediatric patients, and two of the three clinical variables are presented in Table 2. Most family caregivers were women (87%), married (65.5%), housewives (65.5%), and mothers of the patient (77.1%) in addition to having a secondary education (63%) and school-age children (59.2%).

**Table 2:** Sociodemographic Characteristics of the Family Caregivers and Children

Variables	Caregivers		Variables	Children	
	*M* (*SD*)	*n* (%)		*M* (*SD*)	*n* (%)
Sex			Sex		
Female		367 (83)	Female		214 (48)
Male		79 (17)	Male		232 (52)
Age	32.23 (8.65)		Age (months)	32.21 (128.81)	
Marital status			Months of hospitalization	1.71 (1.22)	
Married		179 (40.1)			
Cohabiting		167 (37.4)			
Separated		40 (9)			
Single mother		34 (7.6)			
Divorced		13 (2.9)			
Widowed		6 (1.3)			
Other		7 (1.6)			
Education			Months since diagnosis	3.5 (2.00)	
Illiterate		15 (3.4)			
Primary & secondary		281 (63)			
Preparatory		115 (25.8)			
University		35 (7.8)			
Occupation					
Homemaker		292 (65.5)			
Employed		60 (13.5)			
Merchant		43 (9.6)			
Unemployed		31 (7)			
Laborer		15 (3.4)			
Student		5 (1.1)			
Parental role					
Mother		344 (77.1)			
Father		75 (16.1)			
Sibling		4 (0.9)			
Other		26 (5.8)			
Type of family					
Nuclear		225 (50.4)			
Single parent		74 (16.6)			
Seminuclear		68 (15.2)			
Extended		46 (10.3)			
Other		33 (7.4)			
Family life cycle					
Small children		146 (32.73)			
School-age children		264 (59.2)			
Adult children		35 (7.84)			
Support network					
Family		371 (83.2)			
Institutions		50 (11.2)			
Government		15 (3.4)			
Friends		8 (1.8)			
Income^a^	1.62 (0.93)				
0–3		432 (96.86)			
4–7		13 (2.91)			
More than 8		1 (0.22)			

Note. *N* = 446. *M* = arithmetic mean, SD = standard deviation, *n* = simple absolute frequency, and % = percentage

^a^ Monthly income is approximately 141 US dollars)

The mean time of hospitalization was 1.51 months (95% CI: [1.40–1.62]), and the average time since diagnosis was 3.5 months (95% CI: [3.31–3.69]). Regarding the diagnoses, 330 of the 446 the children (74%) suffered cancer, 31 (7%) had a persistently patent arterial duct, 21 (4.7%) had nephrotic syndrome, 18 (4%) had chronic terminal renal insufficiency, 13 (2.9%) had asthma, 12 (2.7%) had tricuspid atresia, 9 (2%) had Down syndrome, 4 (0.9%) had tetralogy of Fallot, 3 (0.7%) had HIV/AIDS, 3 (0.7%) had a liver or kidney transplant, and 2 (0.4%) had cystic fibrosis.

The average anxiety score among the family caregivers was 14.48 (95% CI: [13.29–15.67], range 0–63, SD = 12.74), with no significant difference between men and women.

### Relationship between caregiver anxiety and the psychosocial, sociodemographic, and clinical variables

Fifteen of the 16 psychosocial variables correlated with caregiver anxiety, and the strength of these associations ranged from low (*r* = 0.102, *p* = 0.031) to high (*r* = 0.526, *p* <  0.001), with a mean (in absolute values) of 0.266 (low). The correlation between the variable self-efficacy beliefs/optimism and caregiver anxiety was not significant. The means, standard deviations, ranges, and *r* values are presented in Table 3.

**Table 3:** Psychosocial Variables for Family Caregivers

Variable/Scale	*M [95% CI]*	*SD*	*Range*	*r*
Anxiety	14.48 [13.29, 15.67]	12.74	0–63	
Personal agency and empowerment	101.26 [100.25, 102.27]	10.80	74–142	0.304^***^
Family support	59.47 [58.53, 60.41]	10.10	17–68	−0.263^***^
Social support	158.66 [156.96, 160.36]	18.26	82–213	−0.196^***^
Self-esteem	41.30 [40.86, 41.74]	4.76	19–59	−0.129^**^
Quality of life	83.63 [82.60, 84.66]	11.05	58–117	−0.313^***^
Family caregiver burden	22.89 [21.77, 24.01]	12.08	0–88	0.487^***^
Depression	13.87 [12.96, 14.78]	9.83	0–55	0.526^***^
Negative coping style (total score)	23.61 [22.77, 24.45]	9.04	0–60	0.268^***^
Insecure attachment (total score)	59.27 [58.29, 60.25]	10.49	25–96	0.241^***^
Parental stress	32.52 [31.76, 33.28]	8.18	17–73	0.244^***^
Self-efficacy beliefs and optimism	20.75 [20.45, 21.05]	3.23	9–36	0.086^ns^
Familism	53.80 [52.17, 55.43]	17.54	18–90	0.102^*^
Emotional well-being index	18.19 [17.71, 18.67]	5.16	3–30	−0.431^***^
Locus of control (Internal)	76.22 [74.96, 77.48]	13.49	34–114	0.104^*^
HSCPs	49.39 [48.82, 49.96]	6.14	33–66	0.125^**^
Resilience	133.32 [131.76, 134.88]	16.74	49–172	−0.261^***^

Note: *N* = 446; *M* = arithmetic mean; *CI* = confidence interval; *SD* = standard deviation; *r* = correlations with anxiety (BAI) calculated using Pearson’s product-moment correlation coefficient; a two-tailed significance test was used to test H_0_: *r* = 0; ^***^ *p* <  0.001. ^**^ *p* <  0.01. ^*^ *p* <  0.05. ^ns^ *p* > 0.05

Anxiety was independent of the four sociodemographic variables of the caregivers (sex, age, education, and family income) as well as the sex (*rbp* = − 0.038, 95% CI: [− 0.130, 0.055], *p* = 0.425) and age of the children (*r* = − 0.076, 95% CI: [− 0.168, 0.017], *p* = 0.108).

Regarding the clinical variables, the child’s hospitalization period had a significant correlation with caregiver anxiety, but its strength of association was trivial (*r =* 0.097, 95% CI: [0.004, 0.188], *p* ≤ 0.05). The correlation between the time since diagnosis and caregiver anxiety was not significant (*r =* 0.018, 95% CI: [− 0.075, 0.111], *p* = 0.705). After eliminating four diagnoses with fewer than five cases, the comparison of means in caregiver anxiety among the remaining seven diagnostic categories was not significant (*F*[6, 427] = 1.127, *p* = 0.270, η^2^ = 0.018; assuming equality of variances by Levene’s test: *F*[6, 427] = 1.583, *p* = 0.150). The correlation was also not significant when dichotomizing the variable “diagnosis” for cancer (0) and another chronic disease (1) (*rbp* = 0.014, 95% CI: [− 0.079, 0.106], *p* = 0.776).

### Multiple linear regression for predicting caregiver anxiety

The 16 correlating variables (15 psychosocial variables and one clinical variable) were introduced as potential predictors of caregiver anxiety. The residual analysis of this first model did not satisfy the normality assumption. The skewness of the residuals was greater than 1, and the coefficient of kurtosis was greater than 2. Consequently, the response variable data were transformed by adding one unit and subsequently obtaining the natural logarithm (Ln [BAI total score + 1]). The analysis of variance showed the overall significance of the new regression model (F [5, 439] = 58.90, *p* <  0.001). This new model was composed of five psychosocial variables as significant predictors (*p*-values lower than 0.05 using a two-tailed t-test). Depression, caregiver burden, emotional well-being, self-esteem, and a negative coping style accounted for 39.5% of the variance in caregiver anxiety. The effect size of the predictors varied from trivial (β = 0.09) to medium (β = 0.31). There was no evidence of multicollinearity among variables, because the VIF values ranged from 1.04 to 1.42 (Table 4). Cook’s distance also did not reveal any problematic data, since the values were all below the cut-off of 1.0. When sorting the data based on their sampling sequence, the Durbin-Watson test revealed that the residuals were independent (DW = 2.08). The scatter diagram of residuals, which was plotted with the standardized predicted values on the x-axis and the standardized residuals on the y-axis, showed homogeneity in the residual variance. The histogram of the residuals had a bell-shaped curve, and the distribution was mesokurtic (*K* = 0.275, 95% CI: -0.178, 0.728) but was slightly skewed to the left (*Sk* = − 0.529, 95% CI: -0.756, − 0.302); therefore, the distribution was approaching normal.

**Table 4:** Regression Model Predicting Anxiety in Family Caregivers

Predictor variables	*B*	*CI B* (95%)		β	*t*	*p*	*VIF*
		Lower bound	Upper bound				
Constant	2.709	1.917	3.501		6.725	< 0.001	
Depression	0.031	0.022	0.039	0.314	7.103	< 0.001	1.429
Caregiver burden	0.019	0.012	0.026	0.239	5.605	< 0.001	1.330
Emotional well-being	−0.036	− 0.051	−0.020	− 0.193	−4.443	< 0.001	1.379
Self-esteem	−0.019	−0.034	− 0.004	−0.094	−2.504	0.013	1.035
Negative coping style	0.009	0.001	0.017	0.087	2.241	0.026	1.114

*Note. N* = 446; *B* = unstandardized coefficients; β = standardized coefficients; *t* = Student’s t-test statistic; *p* = *p*-value for a two-tailed t-test; *VIF* = variance inflation factor. Predicted variable: LN (Anxiety). Method: Backward-elimination

## Discussion

The first objective of the present study was to determine the sociodemographic characteristics and self-reported anxiety levels for family caregivers of pediatric patients with chronic diseases in Mexico. The results showed that the profile of family caregivers of children with complex chronic conditions was characterized by being female, the mother of the patient, young, married, having completed primary or secondary education, working at home, and having a low income. This profile coincides with those described in previous research on family caregivers of children with chronic diseases [14, 49, 50].

In the general US population, Beck et al. [51] reported an average of 15 (*SD* = 11.8) and Osman et al. [52] reported an average of 13.4 (*SD* = 8.9) for the BAI total score. In the general Mexican population, Robles et al. [53] obtained an average that was statistically equivalent to the two US averages (*M* = 12, *SD* = 9). In the present study, the average BAI total score corresponding to family caregivers of children with chronic diseases was statistically equivalent to those in the three aforementioned studies (*M* = 14.48, 95% CI: 13.29–15.67). Importantly, according to the expectation of greater anxiety in the mental health clinic population, the BAI average is significantly higher in the mental health clinic population than in the general population. Steer et al. [54] assessed anxiety using the Beck Anxiety Inventory (BAI) and reported an average BAI score of 25 (SD = 11.4) in a sample of outpatients diagnosed with various types of psychiatric disorders. Vazquez-Morejón et al. [55] reported an average of 26.14 (SD = 13.82) among patients receiving psychiatric treatment in Spain. Therefore, the average among Mexican family caregivers of children with chronic diseases corresponds to the average of the general population.

The second objective of the current study was to examine the relationships among 16 psychosocial variables, six sociodemographic variables, and three clinical variables with anxiety in family caregivers of children with chronic diseases. The results demonstrated that 15 psychosocial variables were significantly associated with family caregiver anxiety. Self-efficacy beliefs/optimism was the only psychosocial variable with no significant correlation with anxiety. In a previous study, this latter variable showed a significant but weak association with caregiver anxiety [21].

The most frequent diagnosis among the studied children was cancer, which represented three-quarters of the sample; the most frequent type of cancer was leukemia (one out of three cases), specifically acute lymphoblastic leukemia (two out of ten cases). The second most frequent diagnosis was the persistently patent arterial duct condition, accounting for seven out of every hundred cases. Interestingly, despite the seriousness of the children’s diagnoses, their caregivers scored low on variables measuring the effects of care (i.e., anxiety, primary caregiver burden, depression, and parental stress) and scored high on variables indicating adjustment to the disease (i.e., personal agency and empowerment, family support, self-esteem, quality of life, positive coping style, attachment style, self-efficacy beliefs and optimism, familism, the emotional well-being index, locus of control, Historic-Psycho-Socio-Cultural-Premises, and resilience). Caregivers also scored low on variables assessing maladjustment (negative coping and insecure attachment). These results coincide with empirical evidence previously reported for this context and population [23, 29, 30, 32, 36, 49].

The anxiety symptomatology in this group of caregivers was positively associated with depression and caregiver burden and was negatively associated with quality of life, negative coping, family support, and social support networks. These results coincide with previous empirical findings [1, 2, 4, 11, 13, 14, 21, 24, 34, 35, 37, 39]. Furthermore, the regression model indicated that depression, a heavy caregiver burden, low emotional well-being, low self-esteem, and a negative coping style helped predict the presence of anxiety in family caregivers. Previous studies have provided evidence supporting the relationship between a negative coping style and a tendency towards negative affect and complaining of an excessive burden [14, 16–18, 26].

Concerns could exist because certain items used to assess depression and caregiver anxiety might be common to each questionnaire. However, upon examining the content of the items for each questionnaire, we found no repeated or very similar items in their content. The depression questionnaire is a global evaluation of depressive symptoms, and the caregiver anxiety questionnaire evaluates situations that are faced during the care of persons with chronic diseases.

Sex, age, education, and family income were independent of family caregiver anxiety. In turn, the child’s sex and age were also independent of caregiver anxiety. Although some of these variables have been reported to be significant in most studies with a trivial or small effect size [13, 14, 27, 31], other studies have reported that none of the variables are significant [56]. The sex of the caregiver is usually a significant variable. The findings of the present study raised a question concerning why sex did not impact the anxiety scores among family caregivers similar to the results of previous studies in other countries [18, 57]. The maternal role may serve as a buffer against anxiety in Mexican women caring for a chronically ill child, causing their average level of anxiety to match that of men. On the other hand, the suffering of daughters and sons seems to equally impact their caregivers, which can be attributed to their young age; half of the children were four years or less, two-thirds were six years or less, and three-quarters were nine years or less.

The child’s hospitalization period was the only one of the three clinical variables that showed a significant correlation, although its strength of association was trivial. A longer hospitalization time was associated with family caregiver anxiety, as expected [30]. The time since diagnosis was independent of caregiver anxiety. This latter variable may not reflect the severity of the disease, but a longer hospitalization time does. Finally, we must consider that the type of diagnosis also did not have an impact on the anxiety of the family caregivers in this research. Therefore, the important factor underlying the concerns and anxiety of caregivers is that the children suffer from diseases that require continuous attention for an indefinite period of time and that neglecting them can compromise their life, regardless of their sex, age, or diagnosis.

The present study must be considered in the context of its limitations. First, because this study used nonprobabilistic sampling, its results do not constitute parametric estimates of the study population (caregivers of pediatric patients with chronic diseases). However, because of the independence of the cases and the large sample size, the sample can be considered representative of the population that receives services at the Federico Gómez Children’s Hospital of Mexico, National Institute of Health, in Mexico City.

## Conclusion

This study demonstrated that the average profile of a family caregiver of a child with a chronic disease, three-quarters of whom had cancer, is someone who identifies as a woman and as the mother of the patient; who is young, married, has completed primary or secondary education; who is a homemaker; and who has a low income. The average levels of anxiety in family caregivers of children with a chronic disease in Mexico correspond to those of the general population. Furthermore, sociodemographic variables, such as sex, age, education, and family income, were not predictors of anxiety. The time elapsed since diagnosis and the type of clinical diagnosis of the child did not have a significant impact on caregiver anxiety; the time of hospitalization showed only a trivial association, but most of the psychosocial variables did influence caregiver anxiety. In particular, depression, negative coping, and insecure attachment were noted to be risk factors for anxiety, whereas an internal locus of control, quality of life, emotional well-being, familism, and positive coping styles were identified as having protective effects. Depression, caregiver burden, emotional well-being, self-esteem, and a negative coping style predominated among the psychosocial variables as predictors of family caregiver anxiety, explaining fourth-tenths of the variance.

Finally, based on the results of this study, we suggest using interventions focused on evaluating and treating cases of anxiety, promoting positive coping styles, increasing self-esteem, and developing coping resources in family caregivers that have a child with a chronic disease. Further research should consider the use of longitudinal studies to examine the effect of the associations found in this study. In addition, the independence between the six sociodemographic variables in this research and family caregiver anxiety should be tested in future empirical studies.

## Abbreviations

BAI: Beck Anxiety Inventory
BDI: Beck Depression Inventory
